# Novel endoscopic guidewire—assisted transection for removal of an embedded pediatric foreign body: a case report

**DOI:** 10.1016/j.igie.2025.10.011

**Published:** 2025-10-21

**Authors:** Courtney Svenstrup, Barrett P. Cromeens, Payton M. Miller, Brett J. Hoskins

**Affiliations:** 1 Division of Pediatric Gastroenterology, Hepatology, and Nutrition, Department of Pediatrics, Riley Hospital for Children at IU Health, Indiana University School of Medicine, Indianapolis, Indiana, USA; 2 Division of Pediatric Surgery, Riley Hospital for Children at IU Health, Indiana University School of Medicine, Indianapolis, Indiana, USA

## Abstract

Pediatric foreign body ingestion is common, but endoscopic removal can be challenging when objects become embedded. We report a novel, nonthermal, guidewire-assisted transection technique to release a metallic carabiner anchored at the pylorus/duodenum by a mucosal bridge. A 13-year-old boy with autism presented 2 weeks after ingestion. Radiographs showed the object unchanged in the stomach over serial imaging. Endoscopy revealed the carabiner fixed at the pylorus, where its spring-loaded gate had closed over a mucosal fold, creating a tissue bridge. A 0.035-inch curved-tip guidewire was looped beneath the bridge and tensioned in a reciprocating motion, mechanically transecting the tissue and freeing the object. A vascular sheath protected the upper gastrointestinal tract during manipulation. The resulting mucosal defect was closed with 5 hemostatic clips, and the patient recovered uneventfully with normal fluoroscopic follow-up imaging. This novel approach avoided surgery and illustrates how device-adapted endoscopic strategies can expand therapeutic options in pediatrics.

## Introduction

Foreign body ingestion is a common clinical scenario in pediatrics.^1^, ^2^, ^3^ Although most ingested objects pass spontaneously, endoscopic retrieval is warranted in cases of high-risk materials (eg, button batteries, multiple magnets, or sharp objects), prolonged impaction, or concerning anatomic locations.^1^^,^^2^^,^^4^ The pylorus represents a challenging site for foreign body removal because of its narrow diameter and proximity to the duodenum. Most endoscopic extractions use standard retrieval techniques, such as forceps, snares, or retrieval nets; however, these can be ineffective when the object is partially embedded or anchored by surrounding tissue.^1^^,^^5^

Metallic objects, especially those that are sharp or have complex shapes, can lead to mucosal injury, impaction, and fixation.^1^ For objects with hinged or spring-loaded components, mucosal entrapment within these mechanical features can cause progressive tissue injury and potential fixation of the object in place. In cases of foreign body entrapment, thermal dissection using endoscopic incisional therapy has been used in both children and adults.^6^^,^^7^ Although this technique is beneficial, electrocautery has limitations, such as the risk of arcing, deep-tissue injury, and poor visualization—particularly in thin-walled and anatomically constrained regions like the pylorus and duodenal bulb.^8^, ^9^, ^10^, ^11^

We describe a novel application of a guidewire-assisted mechanical transection to endoscopically divide a mucosal tissue bridge tethering a metallic carabiner at the pylorus in a pediatric patient. This case demonstrates the expanding range of therapeutic options in pediatric endoscopy and the importance of innovative, cross-disciplinary approaches in managing complex foreign body impactions.

## Case description

A 13-year-old boy with autism spectrum disorder initially presented to his pediatrician shortly after ingesting a metallic carabiner. He was clinically stable and monitored as an outpatient. Serial abdominal radiographs demonstrated a 6.6-cm metallic object in the central abdomen without progression over 2 weeks (Fig. 1). Given persistent impaction, he was referred to the emergency department and was admitted for endoscopic management.

**Figure 1:**
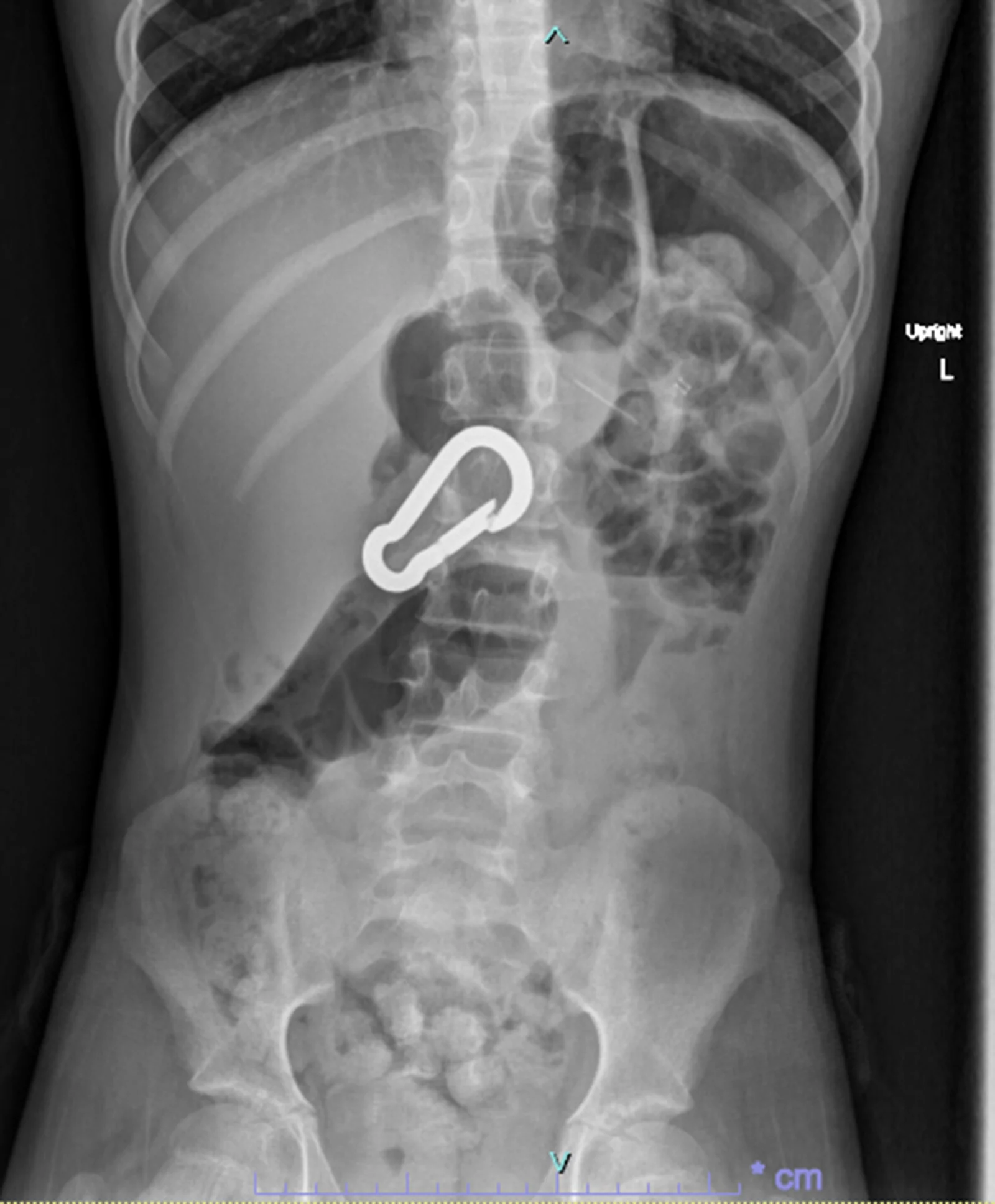
Abdominal radiograph showing a 6.6-cm metallic carabiner in the upper abdomen.

On admission, the patient was stable. Physical examination revealed normal vital signs, no abdominal tenderness, and no obstructive findings. Relevant family and social history were noncontributory. Esophagogastroduodenoscopy performed on hospital day 1 revealed a metallic carabiner impacted at the pylorus, with 1 arm extending into the duodenum (Fig. 2). The spring-loaded gate had entrapped a mucosal fold and gradually eroded through the tissue, resulting in the formation of a tissue bridge over the entrapped segment that anchored the carabiner in place (Fig. 3). Conventional retrieval with forceps and snares was unsuccessful.

**Figure 2:**
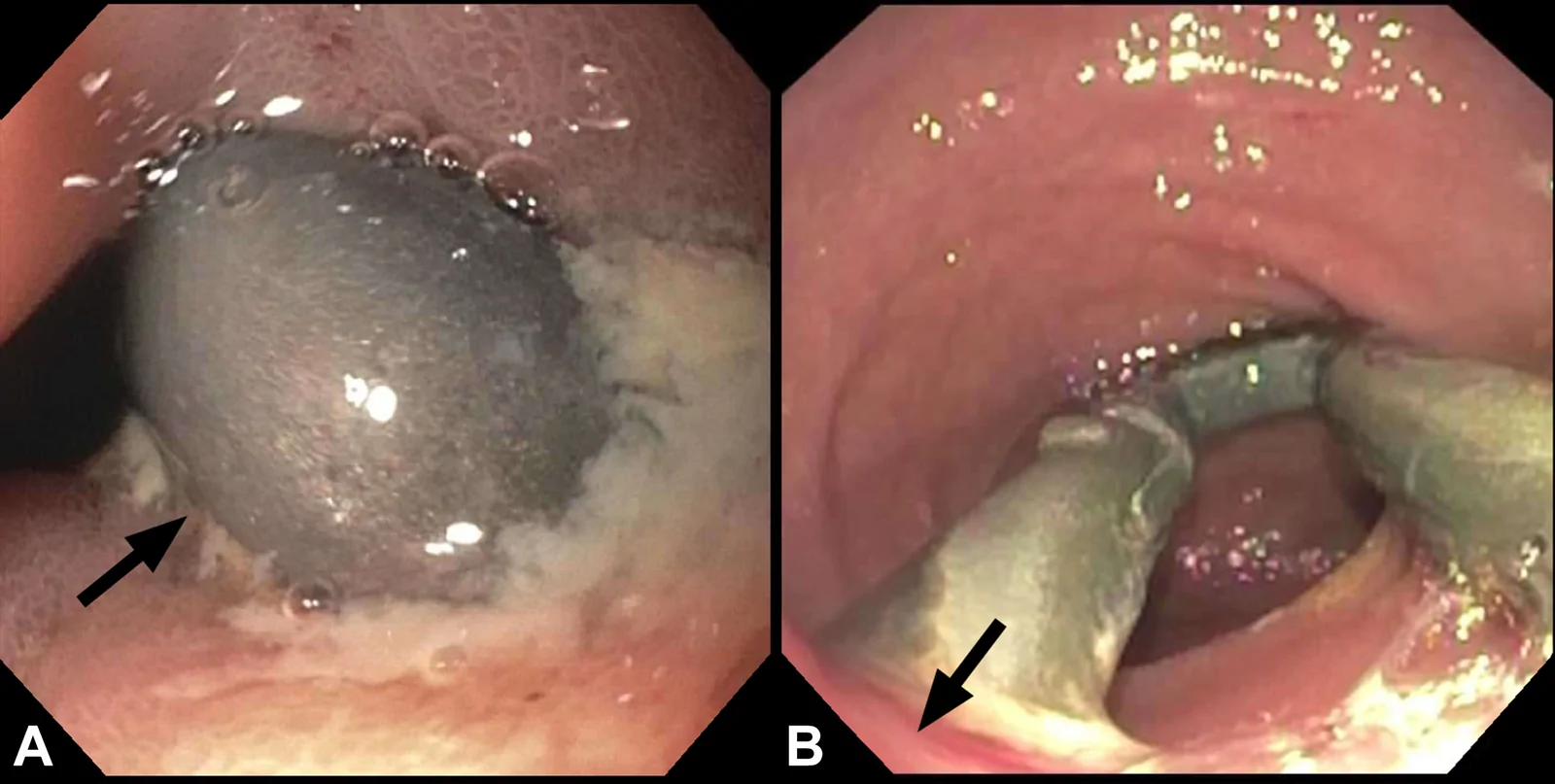
Endoscopic visualization of embedded foreign body. **A,** Carabiner body impacted at the pylorus with surrounding reactive mucosa and overlying exudate. The *arrow* indicates the mucosal tissue bridge created where the spring-loaded gate entrapped a fold and gradually eroded through. **B,** Spring-loaded gate projecting into the duodenum, where it closed around a mucosal fold and reconnected with the carabiner body in the pylorus. The *arrow* highlights the same entrapped fold forming the tissue bridge that anchored the object in place.

**Figure 3:**
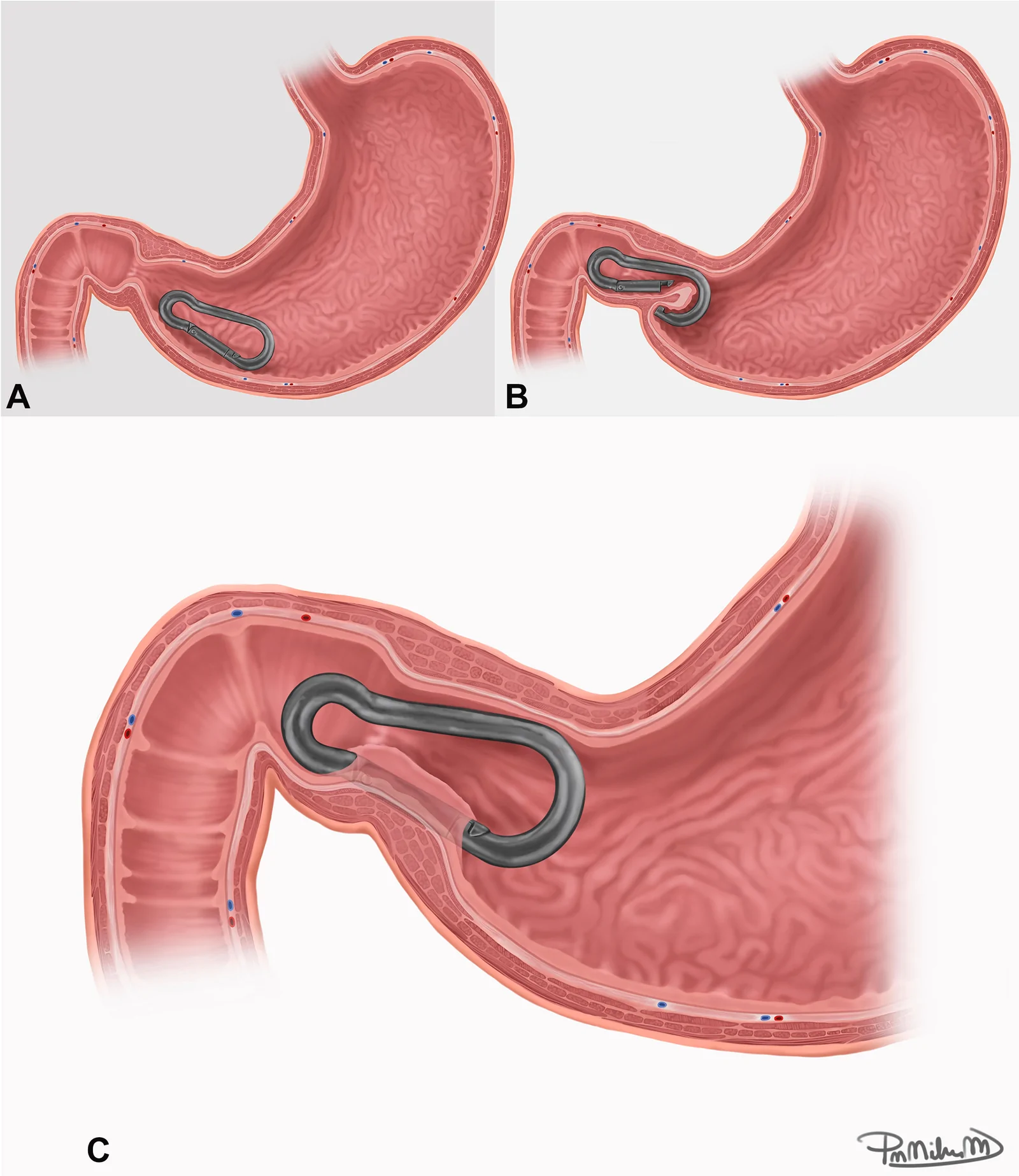
Proposed mechanism of mucosal bridge formation. **A,** Intragastric positioning of the carabiner following ingestion. **B,** Propulsion of the carabiner through the pylorus by gastric peristalsis, during which the spring gate opens. **C,** Entrapment of a mucosal fold within the spring gate with subsequent erosion, forming a tissue bridge tethering the object at the pylorus-duodenal junction.

To avoid surgical intervention, we pursued a mechanical transection strategy in collaboration with pediatric surgery (Figs. 4 and 5). A 0.035-inch Rosen curved-tip guidewire (260 × 1.5 mm; Cook Medical, Bloomington, Ind, USA; Fig. 6A) was advanced endoscopically beneath the tissue bridge and easily passed without resistance. The distal wire was grasped with forceps and pulled back through the esophagus and out the mouth, creating a loop around the tissue bridge. A 10F × 40-cm Flexor Check-Flo vascular introducer sheath (Cook Medical; Fig. 6B) was then threaded over both wire limbs to protect the esophagus and gastroesophageal junction during manipulation. Transection was accomplished in a controlled reciprocating motion under endoscopic visualization and was completed in under 1 minute.

**Figure 4:**
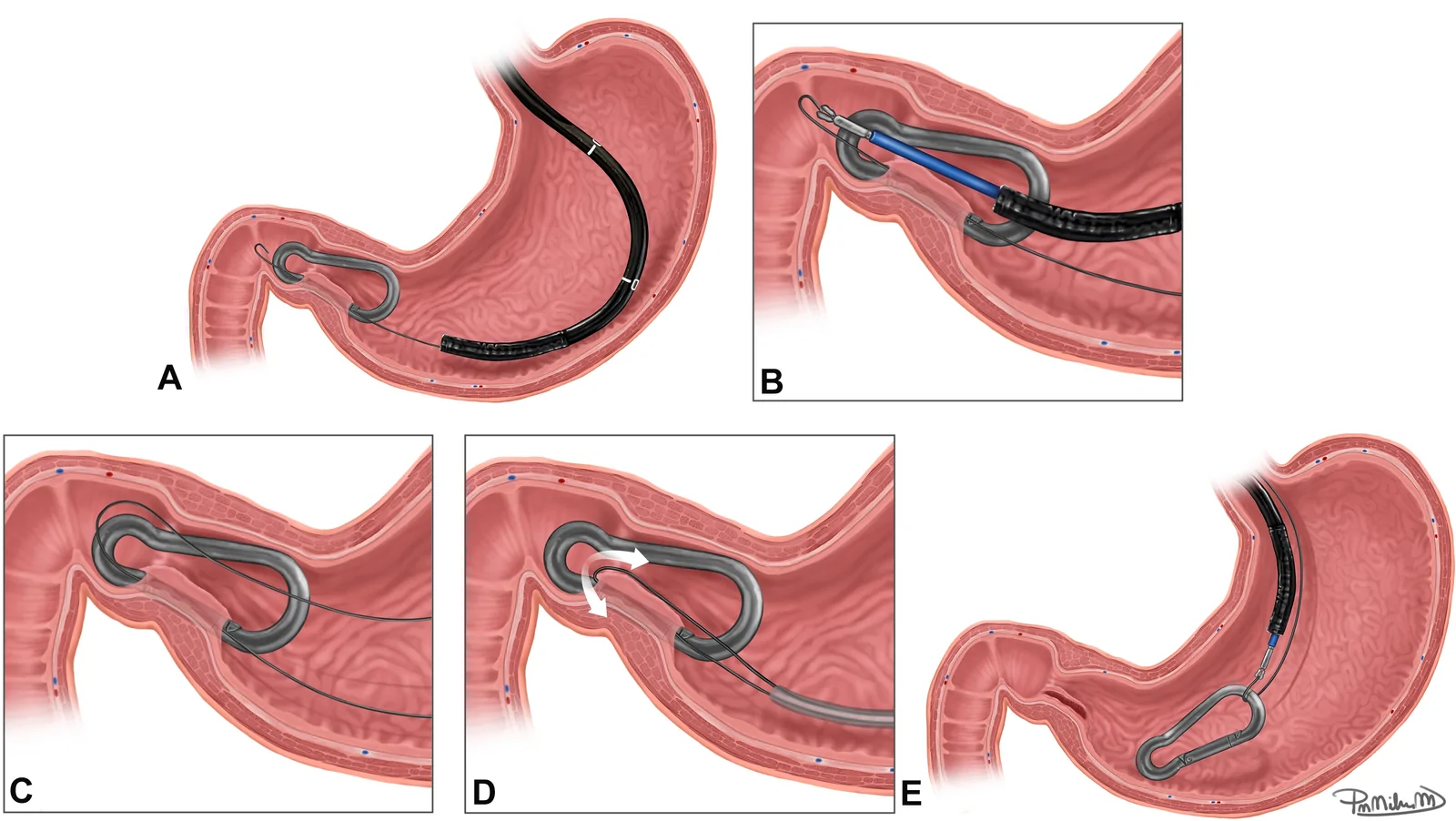
Stepwise schematic of the mechanical (“cheese-wire”) transection technique. **A,** A 0.035-inch curved-tip guidewire is passed endoscopically beneath the mucosal tissue bridge anchoring the carabiner. **B,** The endoscope is withdrawn over the wire and reinserted into the duodenum to retrieve the distal end of the wire with forceps. **C,** The wire is pulled back through the esophagus and out the mouth, creating a loop around the tissue bridge. **D,** A vascular introducer sheath is advanced over both wire limbs to protect the upper gastrointestinal tract. Controlled reciprocating tension is applied to mechanically transect the tissue bridge. **E,** The freed carabiner is extracted endoscopically, and the tissue defect is closed with endoscopic clips.

**Figure 5:**
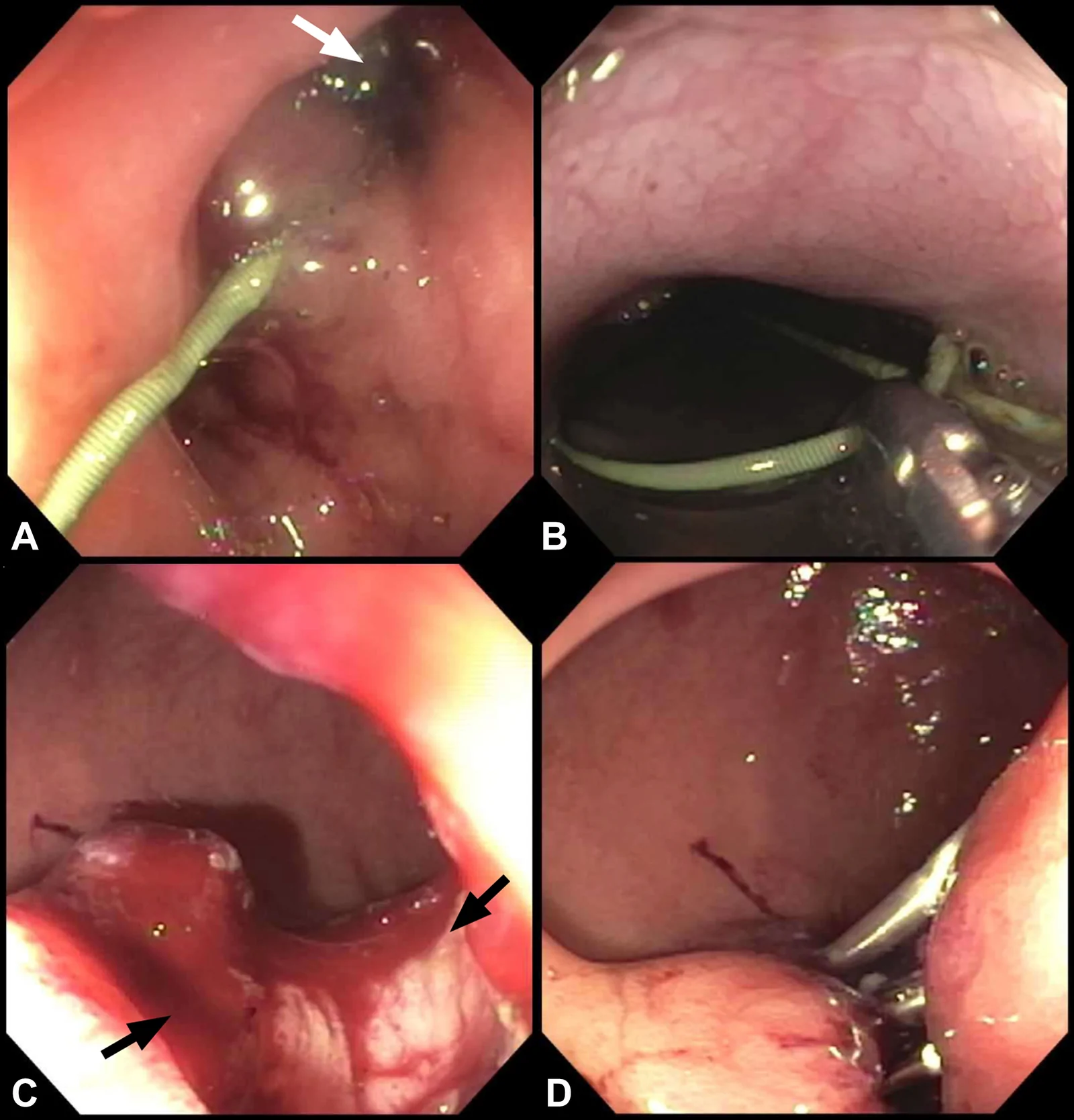
Endoscopic views of wire loop formation, tissue transection, and defect closure. **A,** Guidewire visualized endoscopically after being passed beneath the mucosal tissue bridge. The *white arrow* indicates the visible portion of the carabiner embedded at the pylorus. **B,** Distal end of the guidewire grasped and withdrawn through the esophagus to form a loop around the bridge. **C,** Visible mucosal defect at the pylorus-duodenum junction (*black arrows*) following successful transection and carabiner removal. **D,** Defect closure with endoscopic clips; no bleeding or perforation observed.

**Figure 6:**
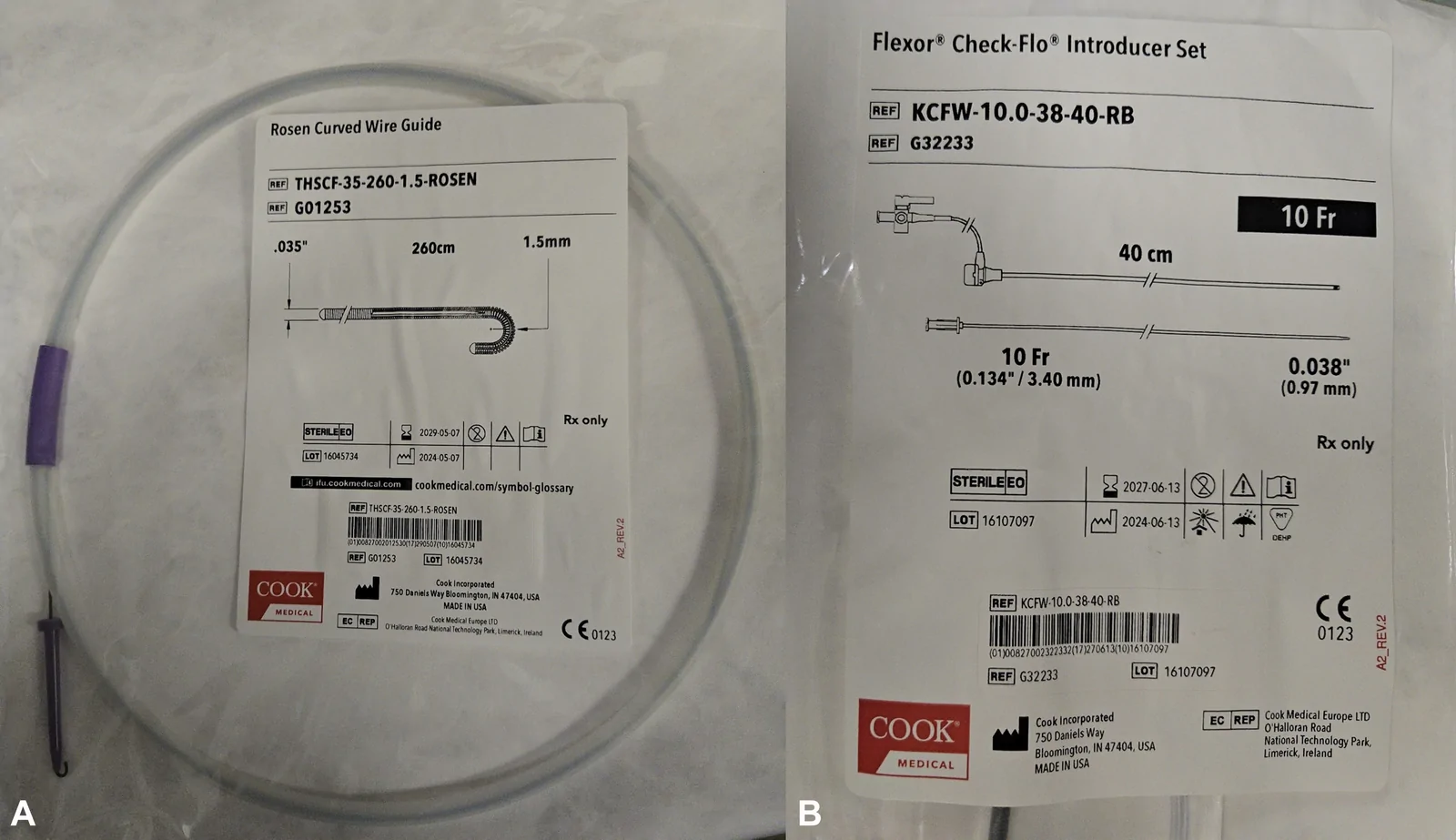
Packaging of devices used for mechanical transection. **A,** Rosen curved-tip guidewire (Cook Medical, Bloomington, Ind, USA). **B,** Flexor Check-Flo introducer sheath (Cook Medical).

The carabiner was successfully released and removed endoscopically. The resulting mucosal defect was closed with 5 hemostatic clips. Postprocedural fluoroscopy confirmed intact luminal integrity without leak or perforation. The patient recovered uneventfully and was discharged the following day. The patient was unable to provide a personal perspective; however, his parents expressed relief at the minimally invasive solution that avoided surgery.

## Discussion

This case illustrates a novel endoscopic approach to a rare but challenging pediatric foreign body scenario: a metallic carabiner embedded at the pylorus/duodenum with partial-thickness mucosal entrapment. The successful use of guidewire-assisted mechanical transection avoided surgical intervention and demonstrates the expanding capabilities of therapeutic pediatric endoscopy.

Foreign body impaction at the pylorus is uncommon, especially in the absence of symptoms like pain, vomiting, or obstructive symptoms.^1^^,^^12^ In this case, the lack of acute clinical signs belied the significant mechanical and mucosal disruption caused by the carabiner. The open-spring gate likely facilitated entrapment of a mucosal fold, forming a tissue bridge that anchored the object in place. This anatomic configuration rendered conventional retrieval methods—like rat-tooth forceps, snares, or nets—ineffective and risked additional mucosal trauma if forceful removal was attempted.

Although thermal incisional therapy using electrocautery is a reasonable option for tissue transection in many settings, the presence of a large metallic surface in direct contact with small-bowel tissue rendered it hazardous in this case. Arcing and thermal injury are of particular concern with monopolar devices in proximity to metal, although bipolar instruments may carry relatively lower risk, as demonstrated in an ex vivo porcine model.^13^ Nonetheless, any thermal approach was judged unsafe within the confined and delicate anatomy of the pylorus and proximal duodenum. In contrast, mechanical transection provided a controlled, nonthermal alternative that allowed gradual division of the tissue bridge using standard endoscopic tools. A curved-tip wire was selected for its atraumatic design, minimizing mucosal injury risk and allowing the tip to recoil toward the scope for controlled positioning. Although not previously reported in endoscopic foreign body cases, to our knowledge, similar “cheese-wire” methods have been described for refractory biliary or pancreatic strictures^14^ and in vascular interventions.^15^^,^^16^ A key adaptation in this case was the use of a vascular introducer sheath to protect surrounding mucosa during wire manipulation, reducing the risk of iatrogenic injury.

Although this method proved effective, it should be reserved for select cases involving tethered foreign bodies where conventional approaches are unsafe or unsuccessful. Successful extraction requires precise wire positioning, loop formation, and sheath control, as well as endoscopic closure of the mucosal defect created during transection. This approach demands endoscopic skills and close interdisciplinary collaboration—particularly with pediatric surgery, who should be immediately available in case of full-thickness injury or failure of endoscopic removal.

This case demonstrates how innovation, adaptability, and early surgical coordination can expand the therapeutic potential of pediatric endoscopy. By repurposing familiar tools like guidewires and vascular sheaths in a novel, coordinated approach, we achieved a minimally invasive solution to an otherwise surgically challenging problem. As pediatric endoscopy continues to advance, such collaborative, device-adaptive strategies may play an increasingly critical role in avoiding operative intervention for complex cases.

## Ethics statement

Institutional Review Board approval was not required for this single-patient case report, which is considered exempt from review under our institutional policy. Written informed consent was obtained from the patient's legal guardian for publication of this case and accompanying images.

## Disclosure

The following author disclosed financial relationships: B. J. Hoskins is a consultant for Mirum Pharmaceuticals, Inc, and 3-D Matrix Inc. All other authors disclosed no financial relationships.
